# Immunization with Live Attenuated *Leishmania donovani* Centrin^−/−^ Parasites Is Efficacious in Asymptomatic Infection

**DOI:** 10.3389/fimmu.2017.01788

**Published:** 2017-12-12

**Authors:** Nevien Ismail, Amit Kaul, Parna Bhattacharya, Sreenivas Gannavaram, Hira L. Nakhasi

**Affiliations:** ^1^Division of Emerging and Transfusion Transmitted Diseases, Center for Biologics Evaluation and Research, US Food and Drug Administration, Silver Spring, MD, United States

**Keywords:** asymptomatic infections, live attenuated vaccines, *Leishmania donovani*, biomarkers of protection, immunogenicity

## Abstract

Currently, there is no vaccine against visceral leishmaniasis (VL). Toward developing an effective vaccine, we have reported extensively on the immunogenicity of live attenuated *LdCentrin*^−/−^ mutants in naive animal models. In VL endemic areas, asymptomatic carriers outnumber symptomatic cases of VL and are considered to be a reservoir of infection. Vaccination of asymptomatic cases represents a viable strategy to eliminate VL. Immunological correlates of protection thus derived might have limited applicability in conditions where the immunized host has prior exposure to virulent infection. To examine whether *LdCen*^−/−^ parasites can induce protective immunity in experimental hosts that have low-level parasitemia from a previous exposure mimicking an asymptomatic condition, we infected C57Bl/6 mice with wild-type *Leishmania donovani* parasites expressing LLO epitope (*LdWT^**LLO**^* 10^3^, i.v.). After 3 weeks, the mice with low levels of parasitemia were immunized with *LdCen*^−/−^ parasites expressing 2W epitope (*LdCen^−/−2W^* 3 × 10^6^ i.v.) to characterize the immune responses in the same host. Antigen experienced CD4^+^ T cells from the asymptomatic (*LdWT^**LLO**^* infected) *LdCen^−/−2W^* immunized, and other control groups were enriched using LLO- and 2W-specific tetramers, followed by Flow cytometric analysis. Our analysis showed that comparable CD4^+^ T cell proliferation and CD4^+^ memory T cell responses (T_CM_) represented by CD62L^hi^, CCR7^+^, and IL-7R^+^ T cell populations were induced with *LdCen^−/−2W^* in both asymptomatic and naive animals that received *LdCen*^−/−^ immunization. Upon restimulation with peptide, T_CM_ cells differentiated into effector T cells and there was no significant difference in the recall response in animals with asymptomatic infection. Following virulent challenge, comparable reduction in splenic parasite burden was observed in both asymptomatic and naive *LdCen*^−/−^ immunized animals concomitant with the development of multifunctional CD4^+^ and CD8^+^ T cells. Further, *LdCen^−/−2W^* immunization resulted in complete clearance of the preexisting asymptomatic infection (*LdWT^**LLO**^*). Our results demonstrate that *LdCen^−/−2W^* immunization could be efficacious for use in asymptomatic VL individuals. Further, immunization with *LdCen*^−/−^ could help in reducing the parasite burden in the asymptomatic cases and aid in controlling the VL in endemic areas.

## Introduction

Visceral leishmaniasis (VL) is a life-threatening vector borne disease caused by *Leishmania donovani* species complex. South East Asia bears most of the global burden of this disease with an estimated 200,000–400,000 new cases reported each year (1). Only a small fraction of all *L. donovani* infections are known to lead to clinically overt disease, with most infections remaining asymptomatic. The reported ratio of asymptomatic infection to active clinical cases of VL ranges from 4:1 in Kenya (2), 6.5:1 to 18.5:1 in Brazil (3), 11:1 in Africa (4), and 9:1 in Indian subcontinent (5). In the absence of definitive longitudinal studies, the reported case conversion rates from an asymptomatic to symptomatic condition range from 1:3.6 to 23.1:100 per year (6, 7). The large divergence in the estimates of asymptomatic cases appears to be due to the limitation of current testing methods that are primarily designed to diagnose clinical disease. Better sampling methods to improve the detection of parasite DNA in asymptomatic carriers continue to be developed (8). The high prevalence rates of asymptomatic cases often complicates the control and elimination of VL since asymptomatic VL cases do not receive any treatment and consequently VL continues to be a public health burden (9). Asymptomatic cases of VL are considered a reservoir for the parasite and can facilitate continued transmission of the disease, even though the role of asymptomatic carriers as reservoirs of the parasites is not clearly understood (10). A recent study of asymptomatic blood donors in Iran showed that human carriers of *Leishmania infantum* in the endemic regions of VL could potentially transmit the parasite through transfusion (11). A prophylactic vaccine could reduce both symptomatic and asymptomatic cases (12).

Chemotherapeutic treatment of VL with different types of drugs has shown success, however such treatment of asymptomatic individuals is not feasible because of the lack of evidence of an overt infection. Due to this limitation, vaccination could be a practical alternative for an effective preventative control of the disease (13). Previous attempts at vaccination based on killed *Leishmania* parasites or defined parasite antigens resulted in limited protection (14). Due to the naturally acquired protection following clinical cure in cutaneous leishmaniasis and VL, a preventative vaccination is considered a practical control measure against leishmaniasis. Live attenuated vaccines allow the host immune system to interact with a wide repertoire of antigens, considered crucial in the development of protective immunity and more importantly cause no pathology (15). Toward developing a live attenuated parasite vaccine, our laboratory has developed several parasite lines with gene deletions (16–18). One of these parasite lines with centrin1 gene deletion from a *L. donovani* strain (*LdCen*^−/−^) has been characterized extensively (15, 16, 19, 20) with the *LdCen*^−/−^ parasites showing 5–8 weeks persistence in mice (15). Our preclinical evaluations demonstrated the safety, immunogenicity characteristics of *LdCen*^−/−^ parasites, and protection against infection with virulent *L. donovani* associated with an induction of a protective adaptive immune response in various animal models including mouse, hamster, and dog (15, 19, 20) and in *ex vivo* studies in human PBMCs in VL endemic areas (21).

However, most of the vaccination studies using live attenuated *Leishmania* parasites have been performed mainly in mice and hamsters that have never been exposed to *Leishmania* or any other microbial pathogens (14). Immunological correlates of protection thus derived might have limited applicability in conditions where the immunized host has prior exposure to virulent infection. In addition, the most likely recipients of the vaccines in the *Leishmania* endemic areas are going to be predominantly asymptomatic carriers of *Leishmania* infection (12). Therefore, it is important to examine whether *LdCen*^−/−^ parasites can induce protective immunity in experimental hosts that have low-level parasitemia resulting from a previous exposure.

Recent developments in enrichment and characterization of epitope-specific T cell populations in experimental infections using tetramer reagents showed that longitudinal studies to track the immunogenicity and development of memory T cell populations are feasible (22, 23). Such studies would enable us to delineate immune responses due to asymptomatic infections and due to immunization with live attenuated parasites. Using methods optimized to enrich epitope-specific CD4^+^ T cell populations, we are able to differentiate the immune responses due to preexisting asymptomatic infection and the vaccine induced response, and identify biomarkers of asymptomatic infection and vaccine induced immunity in the same host. Toward this end, we have prepared recombinant *LdCen*^−/−^ parasites that secrete 2W epitope and *LdWT* parasites that secrete LLO epitope. These epitopes were expressed as fusion proteins with a secretory form of 3′Nucleotidase/nuclease of *L. donovani*. These recombinant parasites (*LdWT^LLO^*/*LdCen^−/−2W^*) were used to infect C57Bl/6 mice and a distinct population of memory cells including central and effector memory cells were identified following immunization. We analyzed the immune responses due to asymptomatic infection and *LdCen*^−/−^ immunization at several time points including after virulent challenge. Our results show that *LdCen*^−/−^ immunization can induce protection even in the hosts with asymptomatic infection as indicated by the reduced parasite burden, development of memory T cell populations, and production of multifunctional T cell response. These studies suggest that it is possible to vaccinate people in the endemic areas where majority of the infected population are asymptomatic carriers of *Leishmania* parasites. Further, development of the novel tools would allow us to assess the immunogenicity and efficacy characteristics of live attenuated parasites such as *LdCen*^−/−^ and address issues of public health importance such as asymptomatic infections toward elimination of VL.

## Materials and Methods

### Mice

The 6- to 8-week-old age-matched female C57Bl/6 mice from Charles River Laboratories were used. All mice were housed in pathogen-free condition in accordance to the guidelines of Food and Drug Administration and National Institutes of Health. The animal protocol for this study has been approved by the Institutional Animal Care and Use Committee at the Center for Biologics Evaluation and Research, US FDA (ASP 1995#26). Further, the animal protocol is in full accordance with “The guide for the care and use of animals as descried in the US Public Health Service policy on Humane Care and Use of Laboratory Animals 2015” (http://grants.nih.gov/grants/olaw/references/phspolicylabanimals.pdf).

### Parasites

The two peptides used in this study were selected based on the frequency of naive precursor CD4^+^ T cell populations and relatively comparable level of expansion following antigenic stimulation *in vivo* reported in previous studies (24). Wild-type *L. donovani* (*LdWT*) expressing LLO peptide 91–99 (NEKYAQAYPNVS), and Centrin1 gene-deleted parasite (*LdCen*^−/−^) expressing 2W1S peptide (EAWGALANWAVDSA) were used. These peptides were fused toward the 3′ end of the *L. donovani* 3′nucleotidase/nuclease and expressed using the *Leishmania* expression plasmid pLexsy-Ble2 (Jena Biosciences). The coding sequences containing the 3′nucleotidase/nuclease and either LLO (pLexsy-Ble2-LLO) or 2W (pLexsy-Ble2-2W) peptides were ligated into Nco I and Not I sites of pLexsy-Ble2 vector. *Leishmania* parasites were transfected with 5 µg of *SwaI* linearized pLexsy-Ble2-LLO or pLexsy-Ble2-2W plasmid DNA. Clones of the recombinant parasites were selected on bleomycin containing Nobel agar plates. For challenge experiments, virulent *L. donovani* (Ld1S) parasites, maintained through serial passages in hamsters, were used. The parasites were cultured according to the procedure described previously (17).

### Immunization and Challenge

Mice were immunized or infected *via* tail vein (i.v.) injection. We have recently shown that immunization or infection *via* i.v. route is comparable to intradermal route with respect to induction of immune response (Banerjee et al., unpublished data). Mice were divided into three groups, the first group (G-I) received 10^3^ stationary phase *LdWT* expressing LLO peptide (*LdWT*^LLO^) and 21 days later the mice were immunized with 3 × 10^6^ *LdCen*^−/−^ expressing 2W1S peptide (*LdCen*^−/−^^2W^). The second group (G-II) received 10^3^ *LdWT*^LLO^ parasites. The third group (G-III) was immunized with 3 × 10^6^ *LdCen*^−/−^^2W^. Age-matched naive mice were used as negative control. In each study, at least four mice were used per group per time point and the experiments were repeated at least twice.

At each experimental time point [11, 21, and 57 days (8 weeks) postimmunization], spleens, inguinal, axillary, cervical, submandibular, and parotid lymph nodes were collected. Single cell suspensions from these organs were used to enrich for 2W- and LLO-specific CD4^+^ T cells using tetramer reagents on Miltenyi LS columns. Enriched cells were labeled and analyzed using flow cytometry. Eight weeks postimmunization, some of the mice received a tail vein injection with a peptide cocktail (2W + LLO, 20 µg each). The mice were euthanized 72 h later and the effector CD4^+^ T cell response was measured.

In some experiments, 10 weeks postimmunization; mice were challenged *via* tail vein injection with 10^5^ virulent *L. donovani* metacyclic parasites. Eight weeks postchallenge, mice were euthanized and parasite load was measured. Immune response following virulent challenge was analyzed by measuring production of proinflammatory cytokines by effector T cells as well as proliferation.

### Tetramers

2W1S:I-A^b^-streptavidin-phycoerythrin (2W-PE) and LLO:I-A^b^-streptavidin-allophycocyanin (LLO-APC) were obtained from NIH tetramer core facility at Emory University, Atlanta, GA, USA.

### Tetramer Enrichment and Flow Cytometry

Tetramer staining was done following the published protocol (25). Spleens and lymph nodes were prepared into a single cell suspension. Cell suspensions were treated with 3 ml of ACK lysis buffer for 5 min at RT. After washing, cells were stained with a cocktail of 2W-PE and LLO-APC tetramers for 1 h in the dark at RT. The cells were labeled with anti-PE and anti-APC magnetic beads for 30 min on ice. Epitope-specific CD4^+^ T cells were enriched on MACS LS magnetic columns for further analysis as described (25). Cells were stained with surface markers on ice with conjugated anti-B220, anti-CD11b, anti-CD11c, and anti-F4/80 all conjugated with eFluor 450; AF700-anti-CD3, BV785 anti-CD4, BV650 anti-CD8α, FITC-anti-CD44, BV605 anti-CD62L, PE-Cy7 anti-IL7R, and PerCP-Cy5.5 anti-CCR7. Cells were then analyzed on an LSR Fortessa (Becton Dickinson). For each sample, >1 million events were recorded at each time point. Data were analyzed with FlowJo software v10 (TreeStar).

### Carboxyfluorescein Succinimidyl Ester (CFSE) Proliferation Assay

The proliferation of antigen experienced T cells following challenge with *LdWT* parasites was assessed by CFSE dilution assay. Age-matched naive mice were used as negative controls. Spleens and lymph nodes harvested from different groups of mice and cell suspension were prepared. Cells were incubated in 5 µM CFSE (Molecular Probes) for 10 min in RPMI 1640 without fetal bovine serum, followed by 5 min of quenching in ice cold RPMI 1640 containing 10% FBS. CFSE stained cells were washed thoroughly before plating in a 96-well tissue culture plates at a concentration of 10^6^ cells/well. Cells were cultured for 7 days at 37°C with 5% CO_2_ in presence of soluble *Leishmania* antigen (SLA, 80 µg/ml). Cells were harvested, washed, and blocked with Fc block (5 µg/ml) for 20 min (4°C) and stained with antimouse CD3 AF700, antimouse CD4 BV785, and antimouse CD8α BV650 (eBioscience, USA) in a 1:200 dilution at 4°C for 30 min. For analysis, single live cells (dead cells were excluded based on staining with the LIVE/DEAD Aqua dye) were gated for CFSE stained CD4^+^ T cells and the extent of proliferation was calculated. Cells were acquired on an LSR Fortessa (BD Biosciences) equipped with 405-, 488-, 561-, and 640-nm laser lines using FACSDiva 6.1.2 software. Data were analyzed with FlowJo.

### Multifunctional T Cell Response

Multifunctional T cell responses were measured 10 weeks postchallenge. Cells from spleen and lymph nodes were plated in 98-well culture plates and stimulated with *L. donovani* SLA (80 µg/ml) in complete RPMI 1640 medium. Cells were incubated at 37°C in a 5% CO_2_ with 95% humidity for 24 h. Golgistop (BD Biosciences) was added to the cultures 4 h prior to the staining procedure. Cells were then prepared for flow cytometry analysis. Samples were stained with surface markers on ice using AF700-anti-CD3, BV-785 anti-CD4, BV-650 anti-CD8α, FITC anti-CD44, and PerCP-Cy5.5 anti-CCR7. Cells were then fixed and permeabilized using Fixation/Permeabilization solution (BD) following manufacturer’s instructions. Cells were then stained for intracellular cytokines using PE-Cy7 anti-IFN-γ, PE anti-TNF-α, and Pacific Blue anti-IL-2. Cells were acquired on an LSR Fortessa equipped with 405, 488, 561, and 640 nm laser lines using FACSDiva 6.1.2 software. Data were analyzed with FlowJo.

### Measurement of Parasite Burden

Parasite burden was measured at 21 days postimmunization and 8 weeks postchallenge. Parasite burden was measured in the spleen by the limited dilution assay previously described (17).

### Statistical Analysis

Differences between data sets were analyzed by one-way analysis of variance to compare three or more groups and unpaired Student’s *t*-tests to compare differences in means between two groups. Statistical significance was considered when the *p* values were <0.05.

## Results

### Expression of Model Epitopes in Wild-type and *LdCen*^−/−^ Parasites to Track-Specific CD4^+^ T Cell Populations

**Figure 1:**
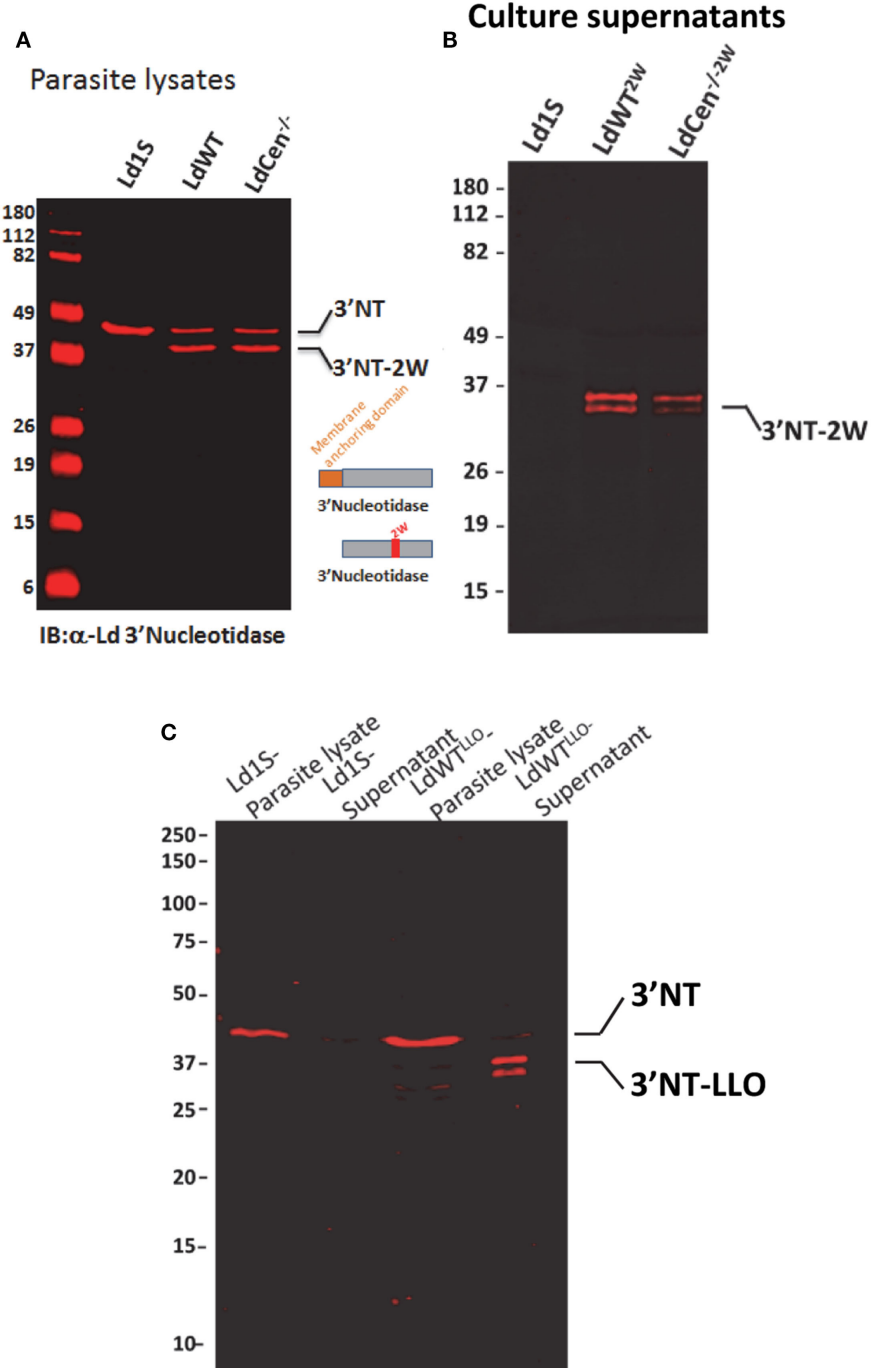
Expression of model epitopes in wild-type and *LdCen*^−/−^ parasites. **(A)** A immunoblot with α-Ld3′Nucleotidase antibody showing the native unmodified 3′Nucleotidase/nuclease and the chimera containing the 2W peptide in the lysates of the recombinant parasites. **(B)** An immunoblot with culture supernatants probed with α-Ld3′Nucleotidase antibody showing the secreted versions of the fusion protein 3′NT-2W. **(C)** An immunoblot with parasite lysates and culture supernatants probed with α-Ld3′nucleotidase antibody showing the secreted versions of the 3′NT-LLO fusion protein.

In order to delineate CD4^+^ T cell immune responses due to virulent wild-type *L. donovani* parasites in an asymptomatic infection and responses due to immunization with *LdCen*^−/−^ parasites in the same experimental host, we modified the two parasite strains to secrete chimeric proteins containing distinct CD4^+^ T cell epitopes. For this purpose, we selected two model epitopes *viz*., LLO peptide (NEKYAQAYPNVS) derived from listeriolysin, produced by the bacterium *Listeria monocytogenes* and 2W1S peptide, a variant of peptide (52–68) from the I-E alpha chain (EAWGALANWAVDSA). The two peptides were selected based on the frequency of naive precursor CD4^+^ T cell populations and relatively comparable level of expansion following antigenic stimulation *in vivo* reported in previous studies (24). The two peptides were expressed as fusion proteins with the *L. donovani* 3′nucleotidase/nuclease lacking the 5′ membrane anchoring domain to ensure secretion of these chimeric proteins. An immunoblot with lysates from these recombinant parasite strains probed with an anti-3′Nucleotidase/nuclease antibody showed the ~45 kDa native 3′nucleotidase/nuclease protein and the ~40 kDa truncated version lacking the membrane anchoring domain but containing the 2W (Figure 1A) or LLO peptides (Figure 1C). Supernatants from these parasite cultures showed only the secreted fusion proteins containing the epitopes (Figures 1B,C).

### A Low-Dose *L. donovani* Infection Mimics Characteristics Reported in Asymptomatic Individuals

**Figure 2:**
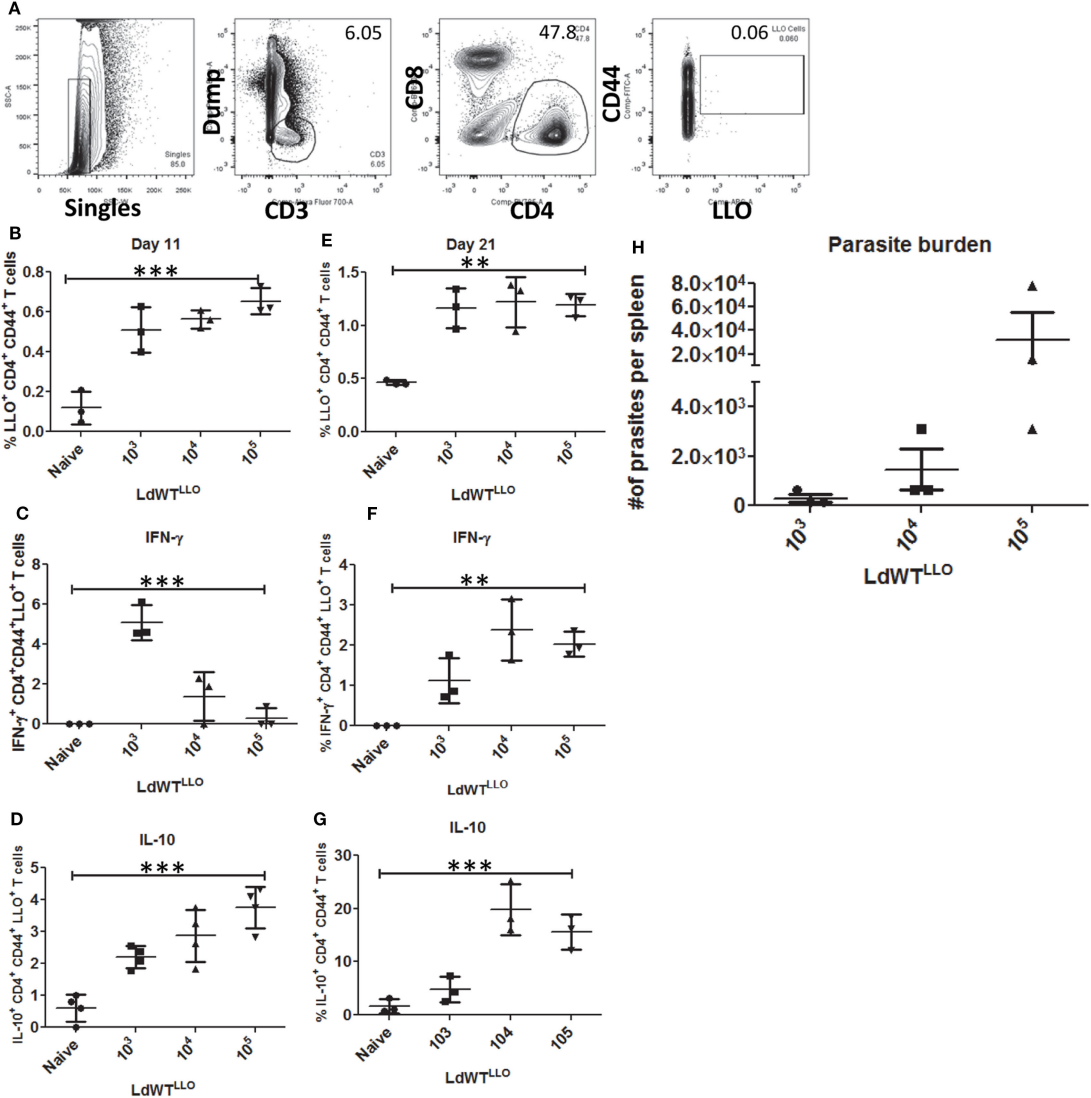
Enrichment and profiling of LLO^+^ CD4^+^ T cells after a low-dose *LdWT^LLO^* infection. **(A)** Gating strategy showing the enrichment of LLO-specific CD4^+^ T cells. CD4^+^ CD44^+^ LLO^+^ T cells are identified as the peptide-specific populations. **(B)** Proliferation of LLO^+^ CD4^+^ T cell populations at 11 days postinfection, **(C)** IFN-γ^+^ CD4^+^ LLO^+^ T cell population at 11 days postinfection **(D)** IL10^+^ CD4^+^ LLO^+^ T cell population at 11 days postinfection. **(E)** Proliferation of LLO^+^ CD4^+^ T cell populations at 21 days postinfection. **(F)** IFN-γ^+^ CD4^+^ LLO^+^ T cell population at 21 days postinfection; **(G)** IL10^+^ CD4^+^ LLO^+^ T cell population at 21 days postinfection. **(H)** Splenic parasite burden at day 21 postimmunization (*LdWT^LLO^*). Data from a representative experiment are shown with three mice per group per time point.

To recapitulate the immunological characteristics of an asymptomatic *L. donovani* infection in a mouse model, we tested various doses of *L. donovani* wild-type parasites expressing LLO epitope (*LdWT^LLO^*) in C57Bl/6 mice. Following intravenous injection of 10^3^, 10^4^, or 10^5^ metacyclic *LdWT^LLO^* parasites, LLO:I-A^b^ tetramer bound CD4^+^ T cell populations were enriched from spleen and lymph nodes on days 11 and 21 postinfection (Figure 2A). Results showed that compared to naive mice, expansion of LLO-specific CD4^+^ T cell populations occurred significantly more in mice that received *LdWT^LLO^* infection. The expansion of LLO-specific CD4^+^ T cell population was evident in all the three doses tested both at days 11 and 21 postinfection (Figure 2B, one-way ANOVA, *p* = 0.0002; Figure S3 in Supplementary Material). Next, we evaluated IFN-γ and IL10 production by LLO:I-A^b^-specific CD4^+^ T cells following the infection to determine the magnitude of these responses in groups of mice that received different doses of *LdWT^LLO^* parasites, and assessed their capacity to produce these cytokines upon restimulation *in vivo* with the LLO peptide. Results showed that the LLO:I-A^b^ CD4^+^ T cells from mice infected with 10^3^ *LdWT^LLO^* parasites produced significantly more IFN-γ compared to naive controls or the mice that were infected with either 10^4^ or 10^5^ LdWT^LLO^ parasites at day 11 postinfection (Figure 2C, one-way ANOVA, *p* = 0.0002; Figure S3 in Supplementary Material). Similarly, the LLO:I-A^b^ CD4^+^ T cells also produced significant amount of IL10 by day 11 p.i. compared to the naive animals, and showed an increasing trend at higher doses of *LdWT^LLO^* infection (Figure 2D, one-way ANOVA, *p* < 0.0001; Figure S3 in Supplementary Material). To confirm if a similar immune response persists in the infected host when the parasite burden goes down, we characterized the LLO:I-A^b^ CD4^+^ T cells isolated on day 21 postinfection (Figure 2E, one-way ANOVA, *p* = 0.001; Figure S3 in Supplementary Material). Results showed that while all groups of mice continued to produce IFN-γ to a comparable degree, only the mice that received 10^3^ *LdWT^LLO^* parasites showed a significantly reduced IL10 expression relative to 10^4^ and 10^5^ parasite infections (Figure 2F, one way ANOVA, *p* = 0.0016 and Figure 2G, one way ANOVA, *p* = 0.0003; Figure S3 in Supplementary Material). Consistently, the splenic parasite burdens showed that 10^3^ group maintained a low level of parasites whereas 10^4^ and 10^5^ groups showed an increasing trend in splenic parasite burden 21 days postinfection (Figure 2H; Figure S3 in Supplementary Material). These results indicated that an infection with 10^3^ *LdWT^LLO^* parasites reproduced conditions typically observed in asymptomatic individuals, including a low level parasite burden, an immune response consisting of predominant IFN-γ and low levels of IL10, indicative of host protection.

### Immunization with *LdCen*^−/−^ in Presence of Low-Dose *LdWT* Infection Induces Significant CD4^+^ T Cell Proliferation

**Figure 3:**
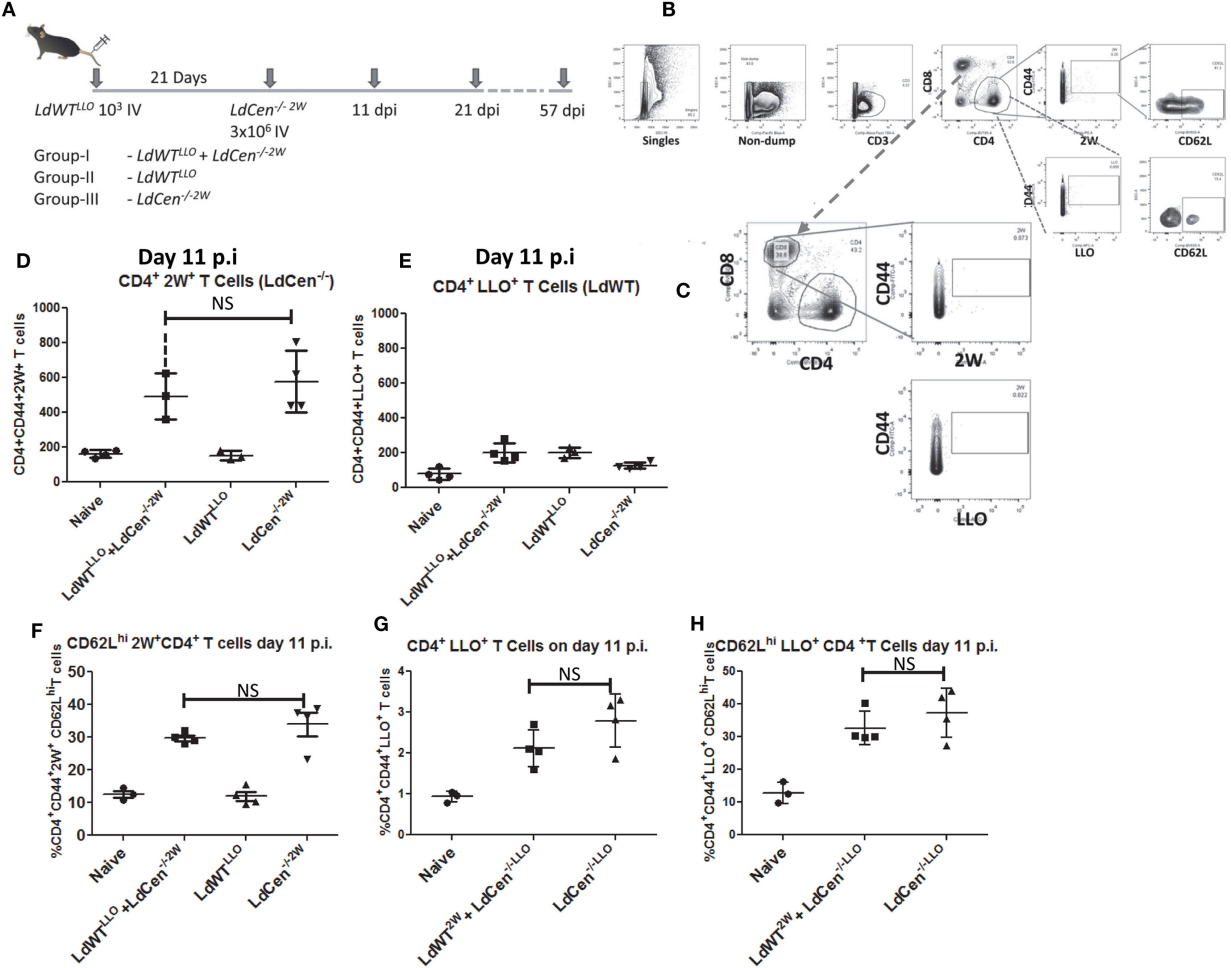
Delineation of the immune responses due to asymptomatic infection (*LdWT^LLO^*) and immunization (*LdCen^−/−2W^*) in the same host. **(A)** Schematic diagram showing C57Bl/6 mice were injected intravenously with 10^3^ *LdWT^LLO^* parasites. The mice were injected with 3 × 10^6^ *LdCen^−/−2W^* parasites 21 days following the first low-dose infection. Immune responses were analyzed at 11, 21, and 57 days postimmunization (p.i.). Various groups of animals that received different regimens of infection are indicated. **(B,C)** Gating scheme to identify 2W^+^-specific and LLO-specific CD4^+^ and CD8^+^ T cell populations. Dump contained markers for B cells, macrophages, DCs, and monocytes. **(D)** Proliferation of 2W^+^ CD4^+^ T cells in the infected mice 11 days postimmunization. **(E)** Proliferation of LLO^+^ CD4^+^ T cells in the infected mice 11 days postimmunization. **(F)** Expression of CD62L on the 2W^+^ CD4^+^ T cells 11 days postimmunization in different groups. **(G)** Converse experiments showing similar proliferation between the two groups (GI and GIII) when the peptide expressed by the respective parasite is swapped at 11 days postimmunization. **(H)** Expression of CD62L on the 2W^+^ CD4^+^ T cells 11 days postimmunization in different groups when the peptide expressed by the respective parasite is swapped. Data from a representative experiment are shown with three to four mice per group per time point.

After establishing the parasitological and immunological conditions representing an asymptomatic infection in a mouse model, we wanted to test whether *LdCen*^−/−^ immunization of these mice with an ongoing low-dose infection could result in a protective response comparable to a naive immunized animal. To this end, we injected 3 × 10^6^ stationary phase *LdCen^−/−2W^* parasites intravenously into mice 21 days after the infection with 10^3^ *LdWT^LLO^* parasites (Figure 3A). Using LLO:I-A^b^ and 2W:I-A^b^ tetramers, we enriched specific CD4^+^ T populations from the cell suspensions prepared from spleen and lymph nodes at several time points following infection(s). Due to the non-competing nature of the 2W and LLO peptides for their cognate T cell receptor, activated LLO^+^ CD4^+^ T cells would only result from *LdWT* infection whereas activated 2W^+^ CD4^+^ T cells would only result from *LdCen*^−/−^ infection. The tetramer staining conditions were optimized to minimize the background binding with either of these tetramers. Following the experimental infections, we first compared the proliferation of antigen experienced CD4^+^ T cells. Results showed that LLO and 2W staining were observed only in the CD4^+^ T cell populations (Figure 3B) but not on the CD8^+^ populations (Figure 3C). Control staining with I-A(b) human CLIP^87–101^ PVSKMRMATPLLMQA (APC-or PE-labeled tetramers) showed lack of binding with the isotype teramers and thus the specificity of 2W and LLO-specific CD4^+^ T cell populations (Figure S1 in Supplementary Material).

### *LdCen*^−/−^ Immunization Enables Induction of Protective Response in Hosts with Low-Dose Asymptomatic Infection with *LdWT*

To test the vaccine induced immunity in presence of preexisting asymptomatic infection, we performed experiments in three different groups of mice. Group I consisted of animals with a preexisting asymptomatic infection that were immunized (*LdWT^LLO^* + *LdCen^−/−2W^*); Group II consisted of animals that received low-dose *LdWT^LLO^* infection alone and Group III consisted of animals that received *LdCen^−/−2W^* immunization alone (Figure 3A). To test the proliferation of the antigen-specific CD4^+^ T cells, we enriched the LLO and 2W peptide-specific populations from spleen and lymph nodes of mice following immunization with *LdCen^−/−2W^* at days 11, 21, and 57 postimmunization (Figure 3A). These time points were chosen to enable analysis of CD4^+^ T cell characteristics in the early phase when the limited replication of *LdCen^−/−2W^* occurs (days 11, 21) and after the clearance of the *LdCen^−/−2W^* parasites due to inherent growth defects and consequently vaccine antigens are no longer persisting in the host (57 days postimmunization). After gating out the non-T cell populations by use of surface markers (Dump; containing macrophage, B cell and DC surface markers), LLO- and 2W-specific activated CD4^+^ T cells were identified based on the staining with the respective tetramers. Results showed that significant proliferation of 2W^+^ CD4^+^ T cells occurred in *LdCen^−/−2W^* (GIII) and in *LdWT^LLO^* + *LdCen^−/−2W^* groups (GI) at day 11 postimmunization compared to naive mice, and the difference between the proliferation was not statistically significant indicating that the underlying asymptomatic infection had no impact on the *LdCen*^−/−^ induced CD4^+^ T cell proliferation (Figure 3D). As expected, only background levels of 2W^+^ CD4^+^ T cells were observed in the *LdWT^LLO^* group (Figure 3D). To test the degree of CD4^+^ T cell proliferation due to low-dose *LdWT^LLO^* infection, we enumerated the LLO^+^ CD4^+^ T cells in the same groups of mice. Results showed that the low-dose infection of *LdWT^LLO^* causes only a limited expansion compared to the naive animals (Figure 3E). It must be noted that this population might represent a steady state CD4^+^ population presumably after a contraction phase since these were sampled 31 days after the initial *LdWT^LLO^* infection (21days of *LdWT^LLO^* + 10days after *LdCen^−/−2W^* infection). The proliferated 2W^+^ CD4^+^ T cells due to *LdCen^−/−2W^* immunization started showing upregulated CD62L expression at day 11 postimmunization indicating that these populations could acquire memory characteristics (Figure 3F). The CD62L populations among the 2W^+^ CD4^+^ T cells were comparable between *LdCen^−/−2W^* alone (GIII) and *LdWT^LLO^* + *LdCen^−/−2W^* (GI) animals (Figure 3F).

To preclude the possibility that the immune responses observed are not biased due to the choice of peptide but specifically due to the infecting parasite, we performed converse experiments with *LdWT* parasites expressing 2W peptide and *LdCen*^−/−^ parasites expressing LLO peptide, respectively. Results showed that proliferation of LLO^+^ CD4^+^ T cells occurred to a similar degree in *LdCen^−/−LLO^* alone and *LdWT^2W^* + *LdCen^−/−LLO^* groups, 11 days postimmunization (Figure 3G). In order to determine if the proliferated CD4^+^ T cells also start to show early memory markers, we measured CD62L expression on the LLO^+^ CD4^+^ T cells. Results showed that a comparable fraction of LLO^+^ CD4^+^ T cells from *LdCen^−/−LLO^* alone and, *LdWT^2W^* + *LdCen^−/−LLO^* groups showed an upregulated CD62L expression indicating that no substantial difference in the quantity and quality of antigen-specific CD4^+^ T cells occurred due to the choice of peptide expressed on the *Leishmania* parasites (Figure 3H).

### Induction of Memory CD4^+^ T Cell Response by *LdCen*^−/−^ in Presence of Low-Dose *LdWT* Infection

**Figure 4:**
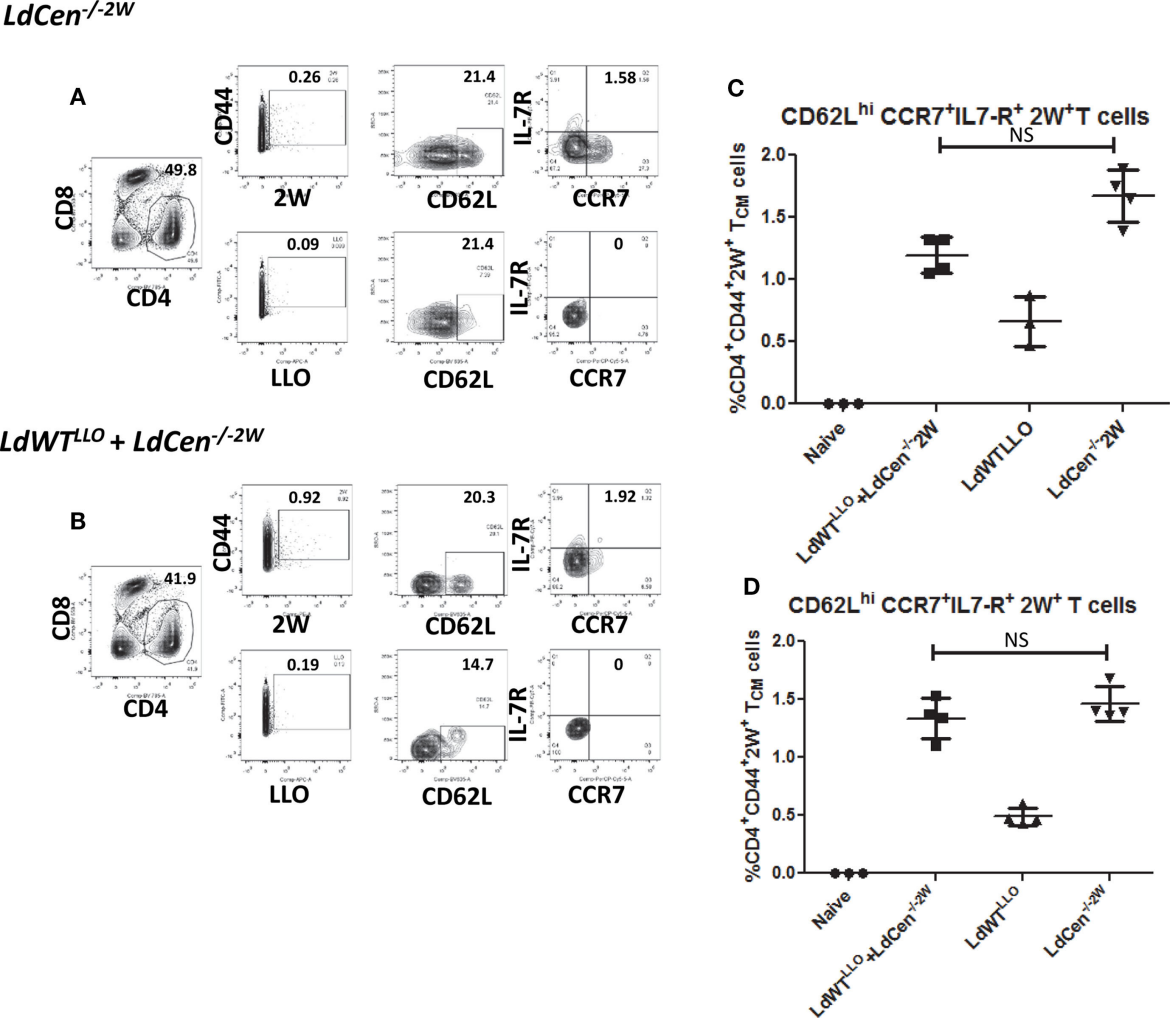
Development of CD4^+^ memory T cell populations in *LdCen^−/−2W^* immunized mice. **(A)** Flow cytometric analysis of 2W^+^ and LLO^+^ CD4^+^ T cell populations enriched from spleen and lymph nodes of immunized animals 21 days after *LdCen^−/−2W^* immunization. CD62L^hi^, IL7-R^+^, CCR7^+^ CD4^+^ T cells are identified as central memory T cell populations. **(B)** Flow cytometric analysis of 2W^+^ and LLO^+^ CD4 T cell populations enriched from spleen and lymph nodes of immunized animals 21 days after *LdCen^−/−2W^* immunization with asymptomatic infection (*LdWT^LLO^* + *LdCen^−/−2W^*). **(C)** CD62L^hi^, IL7-R^+^ CCR7^+^ CD4^+^ T cell populations in different groups of animals at 21 days postimmunization. **(D)** CD62L^hi^, IL7-R^+^ CCR7^+^ CD4^+^ T cell populations 57 days postimmunization with *LdCen^−/−2W^*in various groups. Data from a representative experiment are shown with three to four mice per group per time point.

After establishing the early proliferation of antigen-specific CD4^+^ T cell populations due to *LdCen^−/−2W^* immunization either in the absence or presence of previous asymptomatic infection, we wanted to determine whether the proliferated cells start to acquire memory characteristics by examining the expression of memory markers on the surface (CD62L, CCR7, and IL7R, characteristic of central memory T cells). For this purpose unstimulated CD4^+^ T cells enriched from spleen and lymph nodes were stained with CD44, CD62L, CCR7, and IL7R markers on different time points postimmunization. Results showed that the expanded 2W-specific CD4^+^ T populations start to express CD62L marker as early as day 11 postimmunization (Figure 3F) and continue to express at 21 days postimmunization (Figures 4A,B, CD62L panels). Further, at day 21 postimmunization, the expression of CCR7 was also evident in these CD4^+^ T cell populations indicating that central memory T cell responses are induced due to *LdCen^−/−2W^* parasites (Figures 4A,B, IL7-R and CCR7 panels). Interestingly, no significant difference was observed in these responses between *LdCen^−/−2W^* alone (GIII) and those with preexisting asymptomatic infection (GI, *LdWT^LLO^* + *LdCen^−/−2W^*) at day 21 postimmunization indicating that underlying asymptomatic infection does not adversely impact the immunogenicity of *LdCen^−/−2W^* parasites (Figure 4C). To further test whether the memory CD4^+^ T cell populations could be maintained in the host in the absence of antigen persistence, we examined the vaccine induced responses 57 days (8 weeks) postimmunization. Previous studies using *LdCen*^−/−^ parasites have established that between 5 and 7 weeks the parasites are cleared from the host (15). Results showed that a distinct central memory 2W^+^ CD4^+^ T cell population is maintained in the immunized hosts even in the absence of replicating *LdCen*^−/−^ parasites. No significant difference was observed in the memory populations between groups I and III at this time point again indicating that underlying asymptomatic infection in Group I mice does not diminish the *LdCen^−/−2W^* vaccine induced immunity (Figure 4D).

### Recall Response from the Memory CD4^+^ T Cell Pools

**Figure 5:**
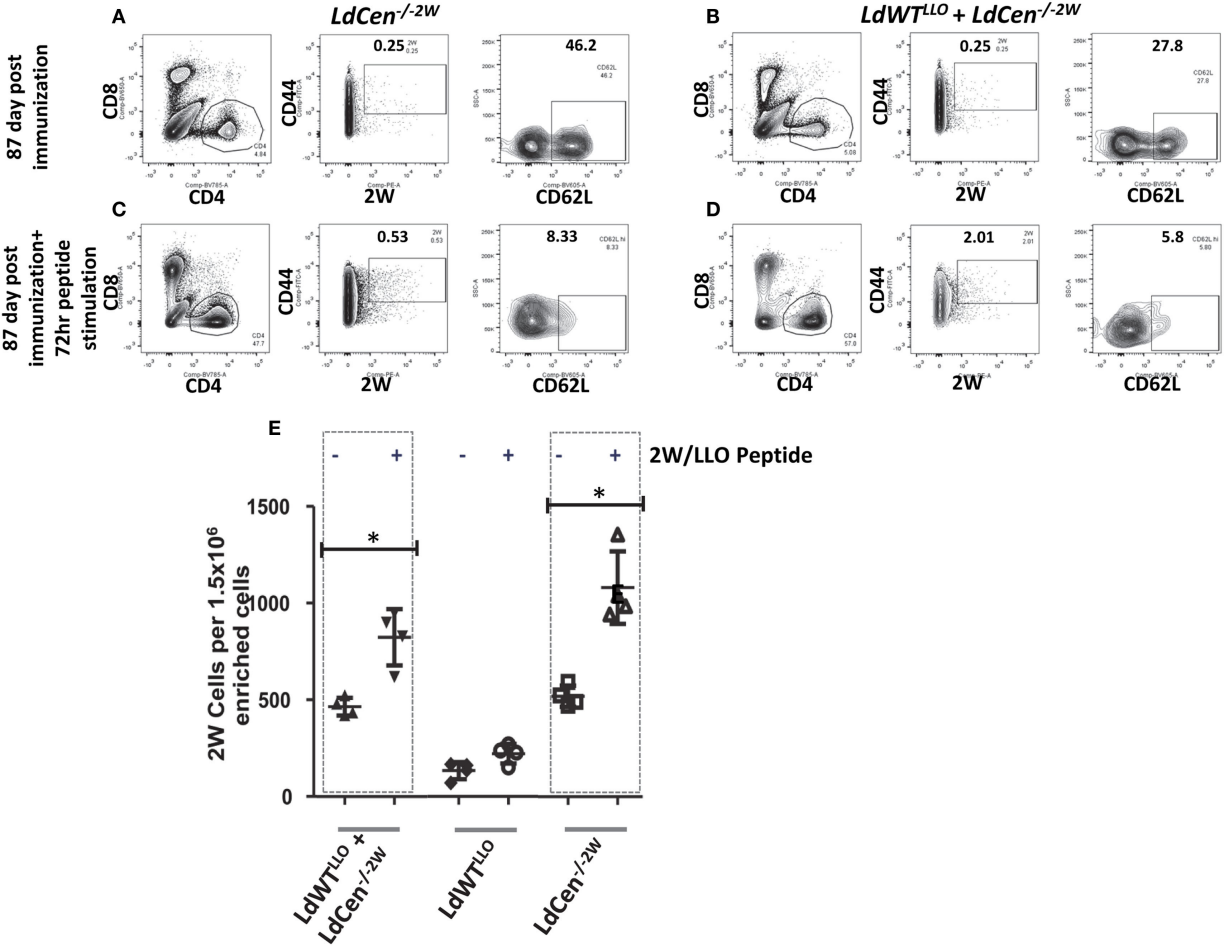
Recall response from the memory CD4^+^ populations after peptide stimulation. **(A)** Flow cytometric analysis of 2W^+^ CD4^+^ CD62L^hi^ populations enriched from spleen and lymph nodes in mice after 12 weeks of immunization with *LdCen^−/−2W^* and **(B)** *LdWT^LLO^* + *LdCen^−/−2W^* immunized group. **(C)** Flow cytometric analysis of 2W^+^ CD4^+^ CD62L^hi^ populations enriched from spleen and lymph nodes in mice after 12 weeks of immunization and after 72hr peptide injection *in vivo* in *LdCen^−/−2W^* immunized and **(D)** *LdWT^LLO^* + *LdCen^−/−2W^* immunized groups. **(E)** Enumeration of 2W^+^ or LLO^+^ CD4^+^ T cells before and after peptide stimulation in various groups. Data from a representative experiment are shown with three to four mice per group.

To test whether the memory CD4^+^ T cell pools induced upon immunization with *LdCen^−/−2W^* parasites maintain the potential to undergo differentiation upon re-exposure to the peptide, we injected the mice with a cocktail containing LLO and 2W peptides intravenously 12 weeks after the immunization. This time point was chosen to ensure that there were no *LdCen^−/−2W^* parasites are persisting in the mice. Following 72 h of stimulation, spleen and lymph nodes were harvested from the mice and the populations of 2W^+^ CD4^+^ T cells were estimated. Results showed that significant proliferation of 2W^+^ CD4^+^ T cells occurred in groups I and III upon restimulation with the 2W peptide compared to unstimulated response in the respective groups (Figures 5A–D) suggesting that the CD4^+^ T cell populations sampled maintain hallmarks of memory characteristics. Interestingly, there was a significant down regulation of CD62L expression following restimulation with 2W peptide in the CD4^+^ T cell populations indicating that an effector response is initiated upon restimulation with the peptide (Figures 5A–D, CD62L panels). The proliferation of 2W^+^ CD4^+^ T cell populations in groups I and III mice was comparable following peptide stimulation *in vivo* indicating that an effector response could also result in mice with low-dose asymptomatic infection (Figure 5E). Taken together, these results indicate that immunization with *LdCen^−/−2W^* induces robust central memory CD4^+^ T cells that are maintained in the absence of persisting parasites, and give rise to effector T cell populations upon restimulation with the cognate peptide. Further a preexisting asymptomatic infection has minimal impact on the immunogenicity of the *LdCen*^−/−^ parasites.

### Induction of Multifunctional CD4^+^ T Cell Response upon Virulent Challenge in the Presence of Preexisting *LdWT* Infection

**Figure 6:**
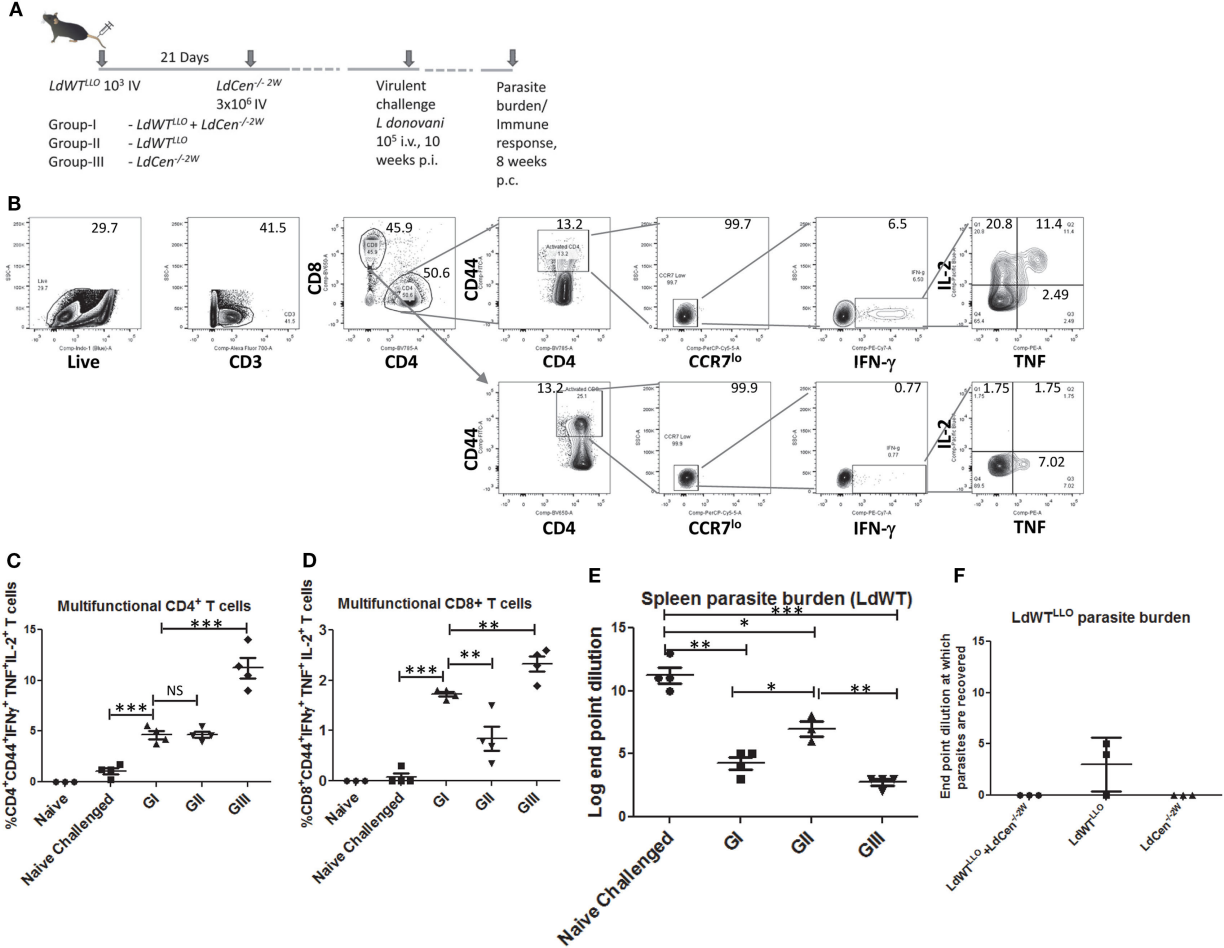
Reduction in parasite burden following virulent challenge in various groups. **(A)** Schematic diagram showing C57Bl/6 mice were injected intravenously with 10^3^ *LdWT^LLO^* parasites. The mice were injected with 3 × 10^6^ *LdCen^−/−2W^* parasites 21 days following the first low-dose infection. At 10 weeks postimmunization all the three groups were challenged with virulent *L. donovani* 10^5^ i.v. Eight weeks postchallenge parasite burden and immune response was measured. **(B)** Gating strategy showing the identification of multifunctional CD4 and CD8 T cells following *in vitro* stimulation with SLA. Splenocytes from the immunized challenged and control mice were stimulated with SLA. Live, activated CD4^+^ or CD8^+^ T cells with low CCR7 expression and producing IFN-γ, TNF and IL2 were identified as multifunctional T cells. **(C)** Multifunctional CD4^+^ and **(D)** CD8^+^ T cells from various groups are represented. **(E)** Splenic parasite burden of the LdWT parasites used in challenge experiment from various groups is expressed as log dilution end points. **(F)** Splenic parasite burden from the initial low-dose asymptomatic infection with *LdWT^LLO^* parasites is shown as log dilution end points. Data from a representative experiment are shown with three to four mice per group.

Our previous studies have shown that naive mice and hamsters immunized with *LdCen*^−/−^ parasites acquire protection against virulent challenge. Further, this protection was mediated by a strong induction of multifunctional CD4^+^ and CD8^+^ T cell response that is characterized by simultaneous production of IFNγ, TNF, and IL2 and reduced IL10 (15). To test whether immunization with *LdCen*^−/−^ parasites can similarly induce a protective immunity mediated by a multifunctional T cell response in animals with a preexisting asymptomatic infection, we evaluated the vaccine induced response in such animals (Figure 6A). This also allowed us to evaluate the effector T cell response of broader specificity after *in vitro* restimulation with SLA containing the full repertoire of *Leishmania* antigens rather than a narrow peptide (LLO or 2W)-specific response as was measured in all earlier experiments in the study. For this purpose, immunized mice challenged with virulent metacyclic LdWT parasites (10^5^ i.v., 10 weeks postimmunization) were sacrificed 8 weeks after challenge (Figure 6A). The immune response in the splenocytes was evaluated after *ex vivo* restimulation with SLA. Both CD4^+^ and CD8^+^ effector T cell populations producing IFNγ, TNF, and IL2 simultaneously were identified upon flow cytometric analysis as CD44^+^CCR7^lo^ IFNγ^+^, TNF^+^, and IL2^+^ cells (Figure 6B). Results showed that compared to naive challenged animals, all the groups produced significantly high proportion of multifunctional CD4^+^ T cells (Figure 6C). Importantly, immunized mice with preexisting *LdWT* infection (G-I, Figure 6C) showed significantly high multifunctional CD4^+^ T cells compared to naive challenged mice (Figure 6C). Mice that were infected with low-dose *LdWT^LLO^* (G-II) also showed significantly more multifunctional CD4^+^ T cells compared to naive challenged mice. The highest proportion of multifunctional CD4^+^ T cell populations was observed in *LdCen^−/−2W^* immunized challenged mice (GIII, Figure 6C). A similar multifunctional response was also observed in CD8^+^ T cell populations in all the three immunized groups (GI-GIII, Figure 6D). However, the CD8^+^ T cell response was somewhat reduced in the low-dose *LdWT^LLO^* group compared to the other two immunized groups (Figure 6D). To test whether the T cells in the immunized host have acquired memory characteristics, we performed proliferation studies of CFSE stained splenocytes in presence of soluble *Leishmania* antigen. Results showed that CFSE stained T cells from all groups of the immunized mice showed significantly high proliferation index compared to naive challenged group suggesting that pools of memory CD4^+^ and CD8^+^ T cells were induced following immunization with *LdCen^−/−2W^* (Figure S2 in Supplementary Material). Corresponding to the protective effector T cell response in our studies, significant reduction in the splenic parasite burden was observed in all the immunized challenged groups compared to naive challenged group (Figure 6E). The *LdCen^−/−2W^* immunized mice with preexisting *LdWT* infection (G-I, Figure 6E) showed significant reduction in the parasite burden compared to naive challenged mice suggesting that *LdCen*^−/−^ immunization can induce protective immunity in these hosts and control splenic parasite burden. As suggested by the superior multifunctional CD4^+^ and CD8^+^ T cell response observed in our studies, the naive *LdCen^−/−2W^* immunized group showed the best reduction in the splenic parasite burden (Figure 6E).

To further test whether the protective immunity induced in mice with asymptomatic infection is sufficient to cause clearance of the prior latent experimental infection, we performed separate limiting dilution cultures of splenocytes in presence of the antibiotic Bleomycin that would allow only the *LdWT^LLO^* cells to grow while the *L. donovani* parasites from the challenge dose are selected out. Results showed that in none of the 3 mice *LdWT^LLO^* parasites could be recovered in the immunized group whereas *LdWT^LLO^* parasites from the original latent infection that were not immunized could still be found in two out of three mice (Figure 6F). These results suggested that immunization of asymptomatic carriers with *LdCen*^−/−^ parasites could be beneficial by lowering or eliminating the latent infection in these individuals. Expectedly, no *LdCen^−/−2W^* parasites could be recovered in presence of Bleomycin 18 weeks after immunization as was reported in our previous studies (15), where *LdCen*^−/−^ parasites are shown to persist only up to 6–8 weeks in the immunized hosts (Figure 6F).

## Discussion

Asymptomatic infections of *Leishmania* parasites are quite common in the VL endemic areas around the World (12). Estimates indicate that in different regions of the world the asymptomatic carriers outnumber the active cases of VL. Additionally, in areas where zoonotic transmission of VL occurs, asymptomatic canine VL is common (26). In the absence of overt clinical disease, the asymptomatic carriers are not routinely treated with anti-Leishmanial drugs. Importantly, the potential for emergence of drug resistance against current anti-Leishmanial drugs, if unwisely employed, precludes their widespread use. The asymptomatic individuals are considered to be the reservoir for the parasite in areas such as India, where anthroponotic transmission is the predominant mechanism of parasite dissemination. Therefore, new strategies to target the asymptomatic carriers are required to advance the goals of elimination of VL. Previous studies have suggested that vaccination could be effective against asymptomatic carriers of *L. donovani* (12). Studies showed that while active VL cases in Indian subcontinent showed a mixed IFN-γ/IL10 response, asymptomatic infections did not lead to antigen induced whole blood IL10 response while showing a positive IFN-γ reaction (27). Other studies in asymptomatic individuals have shown increased CD4^+^ T cell numbers and high levels of IFN-γ production by CD8^+^ T cells (28), CD4^+^ T cells producing IFN-γ and IL-5 (29), and high levels of IL-17 and IL-22 upon *in vitro* antigenic stimulation (30). A recent study reported IP-10 as an accurate biomarker of prior exposure to *L. donovani* and *L. infantum* along with IFN-γ, IL-2, and MIG (31). While the immunological characteristics of asymptomatic carriers are studied extensively, clear estimates of parasite burden in the asymptomatic individuals are lacking due in part to the lack of sensitive detection assays. The potential of asymptomatic carriers as reservoirs of the parasites is indicated by the serological tests and in a limited number of cases by the parasite DNA when family members of VL patients are investigated (32). Most human asymptomatic infections are identified based on immunological characters rather than the presence of low parasite burden. Determining low levels of parasite burden in asymptomatic individuals remains a technical challenge. Therefore, the asymptomatic individuals are identified routinely by immunological characters including the ability to produce various cytokines as IL10 and IFNγ. Further studies in experimental hosts following a low-dose LdWT infection to examine IP-10 and MIG as markers of asymptomatic infection shown in recent studies in Banladesh and Spain (31) along with other markers such as IL-17 and IL-22 shown in a Sudanese study (30) would illuminate the underlying immune mechanisms in asymptomatic infections.

In the present study, we established an animal model which mimics asymptomatic infection. Further, we wanted to test whether live attenuated *LdCen*^−/−^ parasites can induce protective immunity in asymptomatic animals similar to naive animals. Our results showed that the C57Bl/6 mouse model with a low-dose (10^3^) LdWT parasite infection sufficiently recaptures the immunological characteristics observed in asymptomatic carriers. Further, 10^3^ LdWT parasite dose used in this study lies within the range of number of parasites delivered by a sandfly bite (33). Yet, a case could be made to test a range of inocula from a few hundred to less than a thousand parasites. Infection with a very low number of parasites (10^2^) would have made recovery of LLO-specific CD4^+^ T cells difficult. Additionally, previous studies have used 10^5^ parasites in order to track epitope-specific CD4^+^ T cell populations (23). Weighed against these considerations, 10^3^ LdWT parasite dose appears to adequately represent an asymptomatic infection. Accordingly, while the CD4^+^ T cells from 10^3^ parasite infection (*LdWT^LLO^*) continued to produce IFN-γ 21 days postinfection, very low levels of IL10 were observed in this group. Other groups of mice with higher doses (10^4^ or 10^5^ *LdWT^LLO^*) infections clearly showed increasing parasite burdens and increasing levels of IL10 at that time point. However, in our low-dose infection model we observed viable parasites (*LdWT^LLO^*) 3 weeks after the infection unlike human asymptomatic carriers where such detection is rare. It should be noted that this low parasite level was associated with high IFNγ/IL10 ratio suggesting that the host acquired some level of protection. Similar type of immune response was observed in Balb/C mice infected with low number of *L. donovani* parasites (10^3^–10^5^) isolated from cutaneous lesions in Sri Lanka (34).

In order to delineate underlying immune responses due to virulent wild-type infection and to *LdCen*^−/−^ immunization in the same host, we selected two independent peptides that enable tracking of two distinct immune responses in the same host. Importantly two considerations *viz*., presence of comparable number of naive precursor populations and similar levels of proliferation upon stimulation with the cognate peptide guided the selection of two peptides. Previous studies showed that LLO and 2W peptide meet these criteria (24). We considered the possibility that the cross-reaction between 2W and LLO tetramers could occur. However, studies showed that the T cell receptor anchor residues in these two epitopes are very distinct (AQYV in LLO and WW in 2W1S) suggesting that the cross-reaction between the tetramer reagents and the T cell populations is unlikely to occur. Further, this combination of peptides was used in other studies as well (22) that showed no cross reactivity. We also considered the possibility that the response observed could be due to the particular epitope used. To address this concern, we performed a converse experiment with *LdWT* parasites expressing 2W and *LdCen*^−/−^ expressing LLO peptides. Results showed that the proliferation at days 11 and 21 following infection was unchanged and showed that the response observed is due to the *LdCen*^−/−^ specific effects but not due to the epitope selected. Following mouse infection with specific recombinant *Leishmania* parasites, phenotypic characterization of the expanded CD4^+^ T cell populations specific to each peptide expressed by virulent *LdWT* parasites or attenuated *LdCen*^−/−^ parasites enabled us to delineate the immune mechanisms associated with each infection. Such analyses could not be done previously since the tools to distinguish the immune responses from two different parasite strains in the same infected host were not available. We have selected 21 days after infection of *LdWT^LLO^* parasites time point to inject *LdCen^−/−2W^* parasites to allow the establishment of asymptomatic infection and its related effects on the host T cell immunity as shown in Figure 2. In the VL endemic areas, asymptomatic infection may persist for long duration. Therefore, testing the immunogenicity of *LdCen*^−/−^ parasites after establishing a long term asymptomatic infection might also be important. However, immunization with *LdCen*^−/−^ parasites three weeks postinfection represents a much stringent test for immunogenicity with all the potentially inhibitory signals induced by the prior LdWT infection still being active. Waiting too long after a low-dose LdWT infection could lead to the establishment of a stable condition consistent with protective immunity in the infected host. It is doubtful if such situation represents a stringent enough test for the immunogenicity for *LdCen*^−/−^ parasites in the context of asymptomatic infection.

We first measured proliferation of specific CD4^+^ T cell populations following immunization with *LdCen^−/−2W^* parasites. Previous studies have shown that early proliferation of antigen-specific cells determined the magnitude of memory T cell responses (35). Results showed that proliferation of 2W^+^-specific CD4^+^ T cell population occurred to a similar degree in both *LdCen*^−/−^ alone or with a preexisting asymptomatic infection suggesting that the early immunogenicity of *LdCen*^−/−^ parasites was not diminished due to asymptomatic infection. Studies with *L. major* in the resistant C57Bl/6 mouse model showed that early in the infection distinct CD4^+^ T cell populations with memory potential emerge. Following resolution of the cutaneous lesion, these populations mediate protection by giving rise to an effector T cell response (36). Similarly IFN-γ^+^ CD4^+^ T cells were observed in studies that tracked 2W epitope-specific populations in a *L. major* resistant mouse model (23). Our results indicated that the proliferated CD4^+^ T cells acquire central memory characteristics as observed by the CD62L, CCR7, and IL7R expression and that these populations continue to be maintained after the *LdCen^−/−2W^* parasites are cleared from the host. Not surprisingly, of all the groups studied, naive *LdCen*^−/−^^2W^ immunized group showed the greatest expansion and highest frequencies of memory cell populations. The memory cell populations observed at 12 weeks postimmunization with *LdCen*^−/−^ parasites indicated the longevity of the memory responses. Interestingly, restimulation with LLO and 2W peptides induced a recall response as observed by the proliferating effector CD4^+^ T cell populations in *LdCen*^−/−^^2W^ and *LdWT^LLO^* + *LdCen*^−/−^^2W^ groups.

Consistent with the induction of memory T cell populations, we have also observed a both CD4^+^ and CD8^+^ multifunctional T cell response following virulent challenge similar to our previous studies (15, 18). In our previous studies with *LdCen*^−/−^ parasites, we have reported high IgG2a/IgG1 ratio, a robust induction of nitric oxide synthase activity upon stimulation with *Leishmania* antigen following challenge in immunized mice in addition to a strong multifunctional CD4^+^/CD8^+^ T cell responses (15). It is widely accepted that a multifunctional T cell response is a strong correlate of protection, and is commonly employed in *Leishmania* vaccine studies (37–39). While the IgG subtypes could be useful markers in situations where reagents and assays are not available to measure T cell immune responses directly (studies in dogs and hamsters), we have attempted to specifically track the antigen experienced CD4^+^ T cells in the current study. Accordingly, we evaluated the multifunctional response as the correlate of protection in our current study. We observed significant reduction in the parasite burden in the spleens of the challenged animals (GI, GII, and GIII) which was consistent with robust multifunctional T cell response. Even though significant difference in the multifunctional response was observed in CD4^+^ and CD8^+^ T cells between *LdCen*^−/−^^2W^ (GIII) and *LdWT^LLO^* + *LdCen^-/-^*^2W^ (GI) groups, there was no significant difference in splenic parasite burdens between these two groups suggesting that once a certain threshold T cells response is achieved it can control parasitemia. However, in both groups there was significantly higher multifunctional T cell response compared to naive challenged group. Protection was also observed in the low-dose *LdWT^LLO^* infected group (GII). This is coincident with a robust multifunctional CD4^+^ T cell response following challenge in this group. It is interesting to note that multifunctional CD8^+^ T cell response in the *LdWT^LLO^* group (GII) was not as robust compared to other groups suggesting that only live attenuated parasites could yield a strong CD8^+^ T cell response. The protection against virulent challenge in low-dose infected animals is not surprising since a low-dose infection is expected to be associated with a protective immunity as was shown in previous studies (34).

We also observed that the parasites from the prior experimental asymptomatic infection with low-dose *LdWT^LLO^* could be potentially eliminated upon immunization with the *LdCen^−/−2W^* parasites. Successful isolation of *LdWT^LLO^* parasites resulting from the asymptomatic infection group (GII) 21 weeks after initial infection (3 weeks of initial infection followed by 10 weeks immunization and 8 week challenge) but not in the *LdWT^LLO^* + *LdCen^−/−2W^* immunized group (GI) indicates that clearance of the prior asymptomatic infection due to *LdCen^−/−2W^* immunization is a feasible outcome. These results suggest that immunization of the asymptomatic individuals with *LdCen*^−/−^ parasites could be beneficial in clearing the parasites from asymptomatic infection. Since the asymptomatic carriers are the likely recipients of immunization in the VL endemic areas due to the estimated large number of such cases relative to active VL cases, successful elimination of parasites from these asymptomatic carriers by immunization with *LdCen*^−/−^ could help advance the goal of VL elimination by curtailing the anthroponotic transmission of the parasites. Further, strong immunogenicity of *LdCen*^−/−^ parasites and the protective immunity in the hosts with prior asymptomatic infection as demonstrated in this study indicates the potential of immunization in reducing the overall disease burden since the asymptomatic carriers in VL endemic areas are not routinely treated with anti-Leishmanial drugs in the absence of clinically overt disease.

## Ethics Statement

The animal protocol for this study has been approved by the Institutional Animal Care and Use Committee at the Center for Biologics Evaluation and Research, US FDA (ASP 1995#26). Further, the animal protocol is in full accordance with “The guide for the care and use of animals as descried in the US Public Health Service policy on Humane Care and Use of Laboratory Animals 2015” (http://grants.nih.gov/grants/olaw/references/phspolicylabanimals.pdf).

## Author Contributions

SG and HN conceived the project. SG and NI designed the experiments. NI, AK, PB, and SG performed the experiments. SG, NI, and HN analyzed the results. SG wrote the first draft of the manuscript. SG, NI, and HN revised the manuscript.

## Conflict of Interest Statement

The authors declare that the research was conducted in the absence of any commercial or financial relationships that could be construed as a potential conflict of interest.

## Supplementary Material

Figure S1 Control staining with isotype tetramers. I-A(b) human CLIP87-101 PVSKMRMATPLLMQA (APC-or PE-labeled tetramers) were used as recommended by NIH tetramer core facility. Spleen and lymph nodes from C57Bl/6 mice infected with LdWTLLO and *LdCen^−/−2W^* 11 days postinfection were harvested and cells were stained with the same antibody panel as described in Figure 3, except the 2W and LLO tetramers which were replaced with the human CLIP87-101 tetramers.

Figure S2 Proliferative capacity of Ag-specific T cells in various groups of mice after 8-week postimmunization with *LdCen^−/−2W^*. Splenocytes were isolated from each naive and immunized mice, stained with CFSE, and stimulated in vitro with FTAg for 5 days. **(A)** Cells were analyzed by flow cytometry, in which CFSE dilution on particular gated cells was used as the readout for Ag-specific proliferation. **(B)** Percentages of gated CD3^+^ proliferated T cells were calculated. Data from a representative experiment are shown with three to four mice per group.

Figure S3 Enrichment and profiling of LLO^+^CD4^+^ T cells after a low dose LdWTLLO infection (additional data from repeat experiments). **(A)** Proliferation of LLO^+^CD4^+^ T cell populations at 11 days post infection. **(B)** IFN‐γ^+^CD4^+^LLO^+^ T cell population at 11 days post infection. **(C)** IL10^+^CD4^+^LLO^+^ T cell population at 11 days post infection. **(D)** Proliferation of LLO^+^CD4^+^ T cell populations at 21 days post infection. **(E)** IFN‐γ^+^CD4^+^LLO^+^ T cell population at 21 days post infection. **(F)** IL10^+^CD4^+^LLO^+^ T cell population at 21 days post infection. **(G)** Splenic parasite burden at day 21 post immunization (LdWTLLO).

## References

[1] Alvar J Velez ID Bern C Herrero M Desjeux P Cano J Leishmaniasis worldwide and global estimates of its incidence. PLoS One (2012) 7:e35671. 10.1371/journal.pone.0035671 22693548 PMC3365071

[2] Schaefer KU Kurtzhals JA Gachihi GS Muller AS Kager PA. A prospective sero-epidemiological study of visceral leishmaniasis in Baringo District, Rift Valley Province, Kenya. Trans R Soc Trop Med Hyg (1995) 89:471–5. 10.1016/0035-9203(95)90070-5 8560511

[3] Evans TG Teixeira MJ Mcauliffe IT Vasconcelos I Vasconcelos AW Sousa Ade A Epidemiology of visceral leishmaniasis in northeast Brazil. J Infect Dis (1992) 166:1124–32. 10.1093/infdis/166.5.1124 1402024

[4] Hailu A Van Baarle D Knol GJ Berhe N Miedema F Kager PA. T cell subset and cytokine profiles in human visceral leishmaniasis during active and asymptomatic or sub-clinical infection with *Leishmania donovani*. Clin Immunol (2005) 117:182–91. 10.1016/j.clim.2005.06.015 16125466

[5] Ostyn B Gidwani K Khanal B Picado A Chappuis F Singh SP Incidence of symptomatic and asymptomatic *Leishmania donovani* infections in high-endemic foci in India and Nepal: a prospective study. PLoS Negl Trop Dis (2011) 5:e1284. 10.1371/journal.pntd.0001284 21991397 PMC3186756

[6] Topno RK Das VN Ranjan A Pandey K Singh D Kumar N Asymptomatic infection with visceral leishmaniasis in a disease-endemic area in Bihar, India. Am J Trop Med Hyg (2010) 83:502–6. 10.4269/ajtmh.2010.09-0345 20810810 PMC2929041

[7] Das VN Siddiqui NA Verma RB Topno RK Singh D Das S Asymptomatic infection of visceral leishmaniasis in hyperendemic areas of Vaishali district, Bihar, India: a challenge to kala-azar elimination programmes. Trans R Soc Trop Med Hyg (2011) 105:661–6. 10.1016/j.trstmh.2011.08.005 21945327

[8] Kirstein OD Abbasi I Horwitz BZ Skrip L Hailu A Jaffe C Minimally invasive microbiopsies: a novel sampling method for identifying asymptomatic, potentially infectious carriers of *Leishmania donovani*. Int J Parasitol (2017) 47(10–11):609–16. 10.1016/j.ijpara.2017.02.005 28455239 PMC5596977

[9] Singh OP Hasker E Sacks D Boelaert M Sundar S. Asymptomatic *Leishmania* infection: a new challenge for *Leishmania* control. Clin Infect Dis (2014) 58:1424–9. 10.1093/cid/ciu102 24585564 PMC4001287

[10] Stauch A Sarkar PR Picado A Ostyn B Sundar S Rijial S Visceral leishmaniasis in the Indian subcontinent: modelling epidemiology and control. PLoS Negl Trop Dis (2011) 5:e1405 10.1371/journal.pntd.0001405 22140589 PMC3226461

[11] Asfaram S Fakhar M Mohebali M Mardani A Banimostafavi ES Ziaei Hezarjaribi H Asymptomatic human blood donors carriers of *Leishmania infantum*: potential reservoirs for visceral leishmaniasis in northwestern Iran. Transfus Apher Sci (2017) 56:474–9. 10.1016/j.transci.2017.06.001 28648574

[12] Das S Matlashewski G Bhunia GS Kesari S Das P. Asymptomatic *Leishmania* infections in northern India: a threat for the elimination programme? Trans R Soc Trop Med Hyg (2014) 108:679–84. 10.1093/trstmh/tru146 25205664

[13] Mutiso JM Macharia JC Kiio MN Ichagichu JM Rikoi H Gicheru MM. Development of *Leishmania* vaccines: predicting the future from past and present experience. J Biomed Res (2013) 27:85–102. 10.7555/JBR.27.20120064 23554800 PMC3602867

[14] Gannavaram S Dey R Avishek K Selvapandiyan A Salotra P Nakhasi HL. Biomarkers of safety and immune protection for genetically modified live attenuated *Leishmania* vaccines against visceral leishmaniasis – discovery and implications. Front Immunol (2014) 5:241. 10.3389/fimmu.2014.00241 24904589 PMC4033241

[15] Selvapandiyan A Dey R Nylen S Duncan R Sacks D Nakhasi HL. Intracellular replication-deficient *Leishmania donovani* induces long lasting protective immunity against visceral leishmaniasis. J Immunol (2009) 183:1813–20. 10.4049/jimmunol.0900276 19592661

[16] Selvapandiyan A Debrabant A Duncan R Muller J Salotra P Sreenivas G Centrin gene disruption impairs stage-specific basal body duplication and cell cycle progression in *Leishmania*. J Biol Chem (2004) 279:25703–10. 10.1074/jbc.M402794200 15084606

[17] Gannavaram S Davey S Lakhal-Naouar I Duncan R Nakhasi HL. Deletion of ubiquitin fold modifier protein Ufm1 processing peptidase Ufsp in *L. donovani* abolishes Ufm1 processing and alters pathogenesis. PLoS Negl Trop Dis (2014) 8:e2707. 10.1371/journal.pntd.0002707 24587462 PMC3930514

[18] Dey R Dagur PK Selvapandiyan A Mccoy JP Salotra P Duncan R Live attenuated *Leishmania donovani* p27 gene knockout parasites are nonpathogenic and elicit long-term protective immunity in BALB/c mice. J Immunol (2013) 190:2138–49. 10.4049/jimmunol.1202801 23338240 PMC3578143

[19] Fiuza JA Gannavaram S Santiago Hda C Selvapandiyan A Souza DM Passos LS Vaccination using live attenuated *Leishmania donovani* centrin deleted parasites induces protection in dogs against *Leishmania infantum*. Vaccine (2015) 33:280–8. 10.1016/j.vaccine.2014.11.039 25475955

[20] Fiuza JA Dey R Davenport D Abdeladhim M Meneses C Oliveira F Intradermal immunization of *Leishmania donovani* centrin knock-out parasites in combination with salivary protein LJM19 from sand fly vector induces a durable protective immune response in hamsters. PLoS Negl Trop Dis (2016) 10:e0004322. 10.1371/journal.pntd.0004322 26752686 PMC4708988

[21] Avishek K Kaushal H Gannavaram S Dey R Selvapandiyan A Ramesh V Gene deleted live attenuated *Leishmania* vaccine candidates against visceral leishmaniasis elicit pro-inflammatory cytokines response in human PBMCs. Sci Rep (2016) 6:33059. 10.1038/srep33059 27624408 PMC5021981

[22] Moon JJ Chu HH Pepper M Mcsorley SJ Jameson SC Kedl RM Naive CD4(+) T cell frequency varies for different epitopes and predicts repertoire diversity and response magnitude. Immunity (2007) 27:203–13. 10.1016/j.immuni.2007.07.007 17707129 PMC2200089

[23] Pagan AJ Peters NC Debrabant A Ribeiro-Gomes F Pepper M Karp CL Tracking antigen-specific CD4+ T cells throughout the course of chronic *Leishmania major* infection in resistant mice. Eur J Immunol (2013) 43:427–38. 10.1002/eji.201242715 23109292 PMC4086308

[24] Nelson RW Beisang D Tubo NJ Dileepan T Wiesner DL Nielsen K T cell receptor cross-reactivity between similar foreign and self peptides influences naive cell population size and autoimmunity. Immunity (2015) 42:95–107. 10.1016/j.immuni.2014.12.022 25601203 PMC4355167

[25] Moon JJ Chu HH Hataye J Pagan AJ Pepper M Mclachlan JB Tracking epitope-specific T cells. Nat Protoc (2009) 4:565–81. 10.1038/nprot.2009.9 19373228 PMC3517879

[26] Otranto D Dantas-Torres F. The prevention of canine leishmaniasis and its impact on public health. Trends Parasitol (2013) 29:339–45. 10.1016/j.pt.2013.05.003 23746747

[27] Singh OP Gidwani K Kumar R Nylen S Jones SL Boelaert M Reassessment of immune correlates in human visceral leishmaniasis as defined by cytokine release in whole blood. Clin Vaccine Immunol (2012) 19:961–6. 10.1128/CVI.00143-12 22539471 PMC3370446

[28] Hailu A Gramiccia M Kager PA. Visceral leishmaniasis in Aba-Roba, south-western Ethiopia: prevalence and incidence of active and subclinical infections. Ann Trop Med Parasitol (2009) 103:659–70. 10.1179/000349809X12554106963555 20030990

[29] Mary C Auriault V Faugere B Dessein AJ. Control of *Leishmania infantum* infection is associated with CD8(+) and gamma interferon- and interleukin-5-producing CD4(+) antigen-specific T cells. Infect Immun (1999) 67:5559–66. 10531200 10.1128/iai.67.11.5559-5566.1999 PMC96926

[30] Pitta MG Romano A Cabantous S Henri S Hammad A Kouriba B IL-17 and IL-22 are associated with protection against human kala azar caused by *Leishmania donovani*. J Clin Invest (2009) 119:2379–87. 10.1172/JCI38813 19620772 PMC2719936

[31] Ibarra-Meneses AV Ghosh P Hossain F Chowdhury R Mondal D Alvar J IFN-gamma, IL-2, IP-10, and MIG as biomarkers of exposure to *Leishmania* spp., and of cure in human visceral leishmaniasis. Front Cell Infect Microbiol (2017) 7:200 10.3389/fcimb.2017.00200 28620584 PMC5449718

[32] Banu SS Meyer W Ahmed BN Kim R Lee R. Detection of *Leishmania donovani* in peripheral blood of asymptomatic individuals in contact with patients with visceral leishmaniasis. Trans R Soc Trop Med Hyg (2016) 110:286–93. 10.1093/trstmh/trw027 27198212 PMC4914877

[33] Kimblin N Peters N Debrabant A Secundino N Egen J Lawyer P Quantification of the infectious dose of *Leishmania major* transmitted to the skin by single sand flies. Proc Natl Acad Sci U S A (2008) 105:10125–30. 10.1073/pnas.0802331105 18626016 PMC2481378

[34] Mccall LI Zhang WW Ranasinghe S Matlashewski G. Leishmanization revisited: immunization with a naturally attenuated cutaneous *Leishmania donovani* isolate from Sri Lanka protects against visceral leishmaniasis. Vaccine (2013) 31:1420–5. 10.1016/j.vaccine.2012.11.065 23219435

[35] Tubo NJ Fife BT Pagan AJ Kotov DI Goldberg MF Jenkins MK Most microbe-specific naive CD4(+) T cells produce memory cells during infection. Science (2016) 351:511–4. 10.1126/science.aad0483 26823430 PMC4776317

[36] Colpitts SL Scott P. The early generation of a heterogeneous CD4+ T cell response to *Leishmania major*. J Immunol (2010) 185:2416–23. 10.4049/jimmunol.1000483 20624946 PMC2944829

[37] Guha R Gupta D Rastogi R Vikram R Krishnamurthy G Bimal S Vaccination with *Leishmania* hemoglobin receptor-encoding DNA protects against visceral leishmaniasis. Sci Transl Med (2013) 5:202ra121. 10.1126/scitranslmed.3006406 24027025

[38] Hofmeyer KA Duthie MS Laurance JD Favila MA Van Hoeven N Coler RN Optimizing immunization strategies for the induction of antigen-specific CD4 and CD8 T cell responses for protection against intracellular parasites. Clin Vaccine Immunol (2016) 23:785–94. 10.1128/CVI.00251-16 27466350 PMC5014921

[39] Coler RN Duthie MS Hofmeyer KA Guderian J Jayashankar L Vergara J From mouse to man: safety, immunogenicity and efficacy of a candidate leishmaniasis vaccine LEISH-F3+GLA-SE. Clin Transl Immunology (2015) 4:e35. 10.1038/cti.2015.6 26175894 PMC4488838

