# Supplementary figures and images for: Immunization with Live Attenuated Leishmania donovani Centrin−/− Parasites Is Efficacious in Asymptomatic Infection
Source: Front Immunol. 2017 Dec 12;8:1788. doi: 10.3389/fimmu.2017.01788 (PMC5732910; doi:10.3389/fimmu.2017.01788)

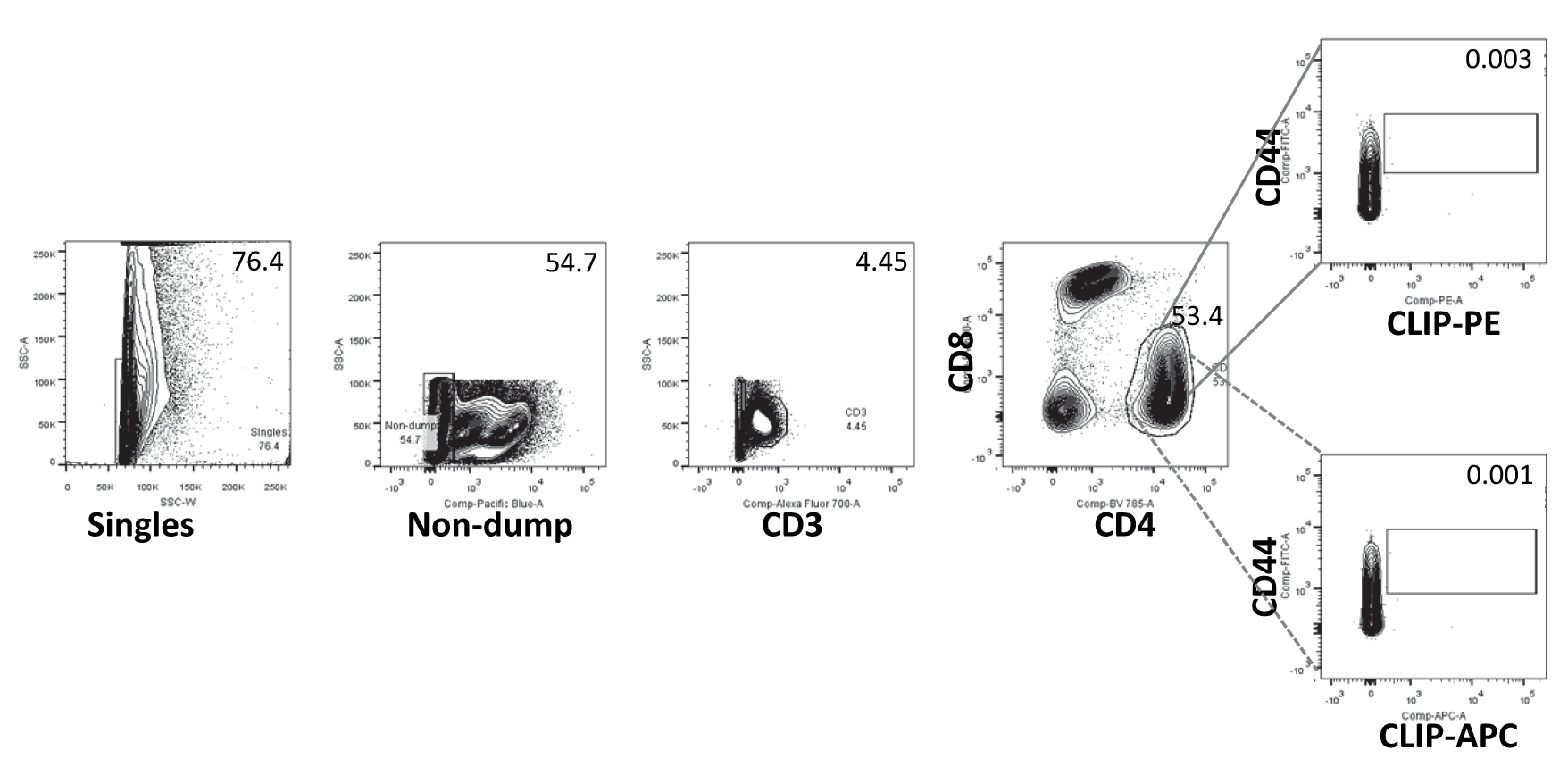

Supplement: Figure S1 — Control staining with isotype tetramers. I-A(b) human CLIP87-101 PVSKMRMATPLLMQA (APC-or PE-labeled tetramers) were used as recommended by NIH tetramer core facility. Spleen and lymph nodes from C57Bl/6 mice infected with LdWTLLO and LdCen−/−2W 11 days postinfection were harvested and cells were stained with the same antibody panel as described in Figure 3, except the 2W and LLO tetramers which were replaced with the human CLIP87-101 tetramers. [file Image_1.TIF]

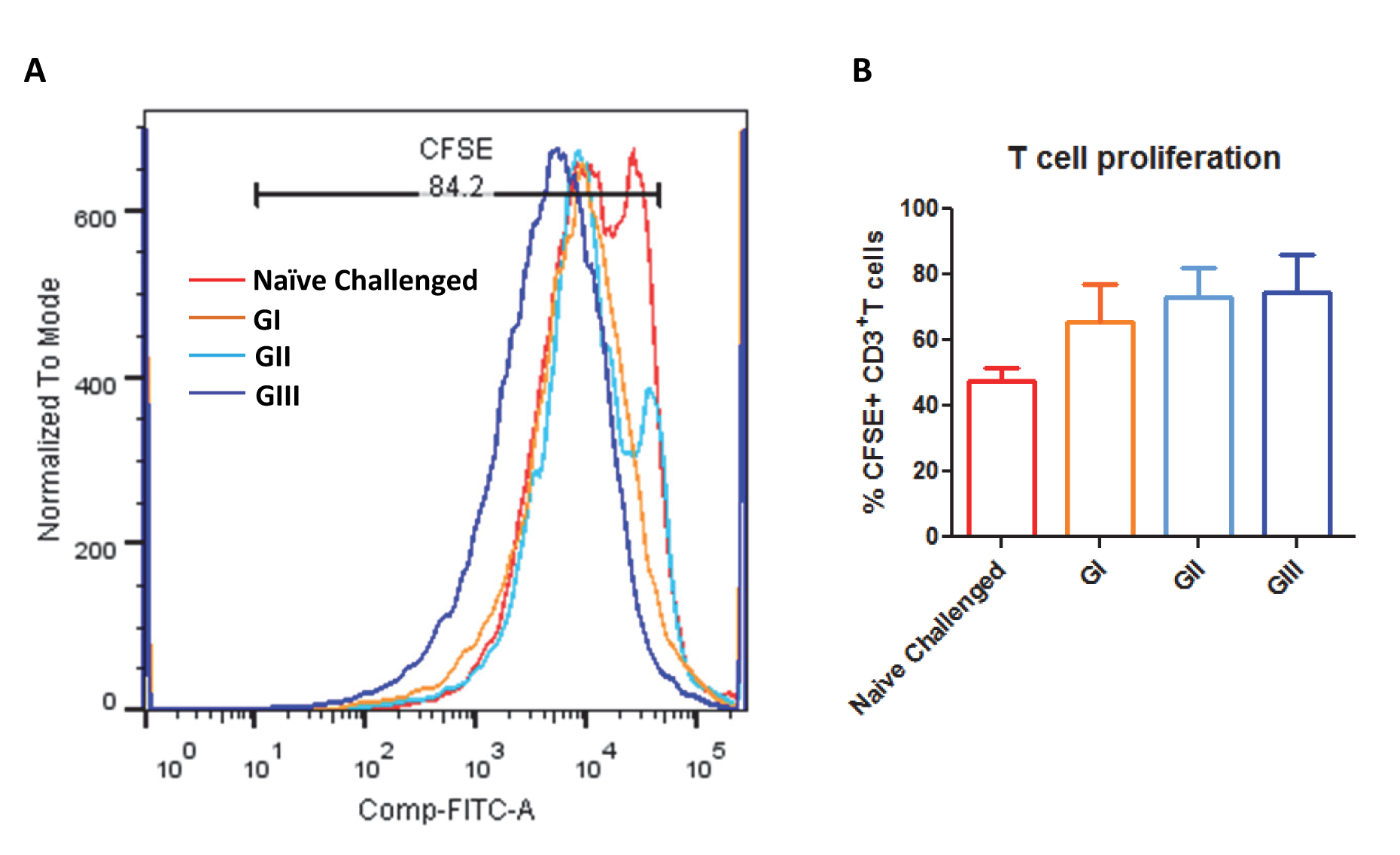

Supplement: Figure S2 — Proliferative capacity of Ag-specific T cells in various groups of mice after 8-week postimmunization with LdCen−/−2W. Splenocytes were isolated from each naive and immunized mice, stained with CFSE, and stimulated in vitro with FTAg for 5 days. (A) Cells were analyzed by flow cytometry, in which CFSE dilution on particular gated cells was used as the readout for Ag-specific proliferation. (B) Percentages of gated CD3+ proliferated T cells were calculated. Data from a representative experiment are shown with three to four mice per group. [file Image_2.TIF]

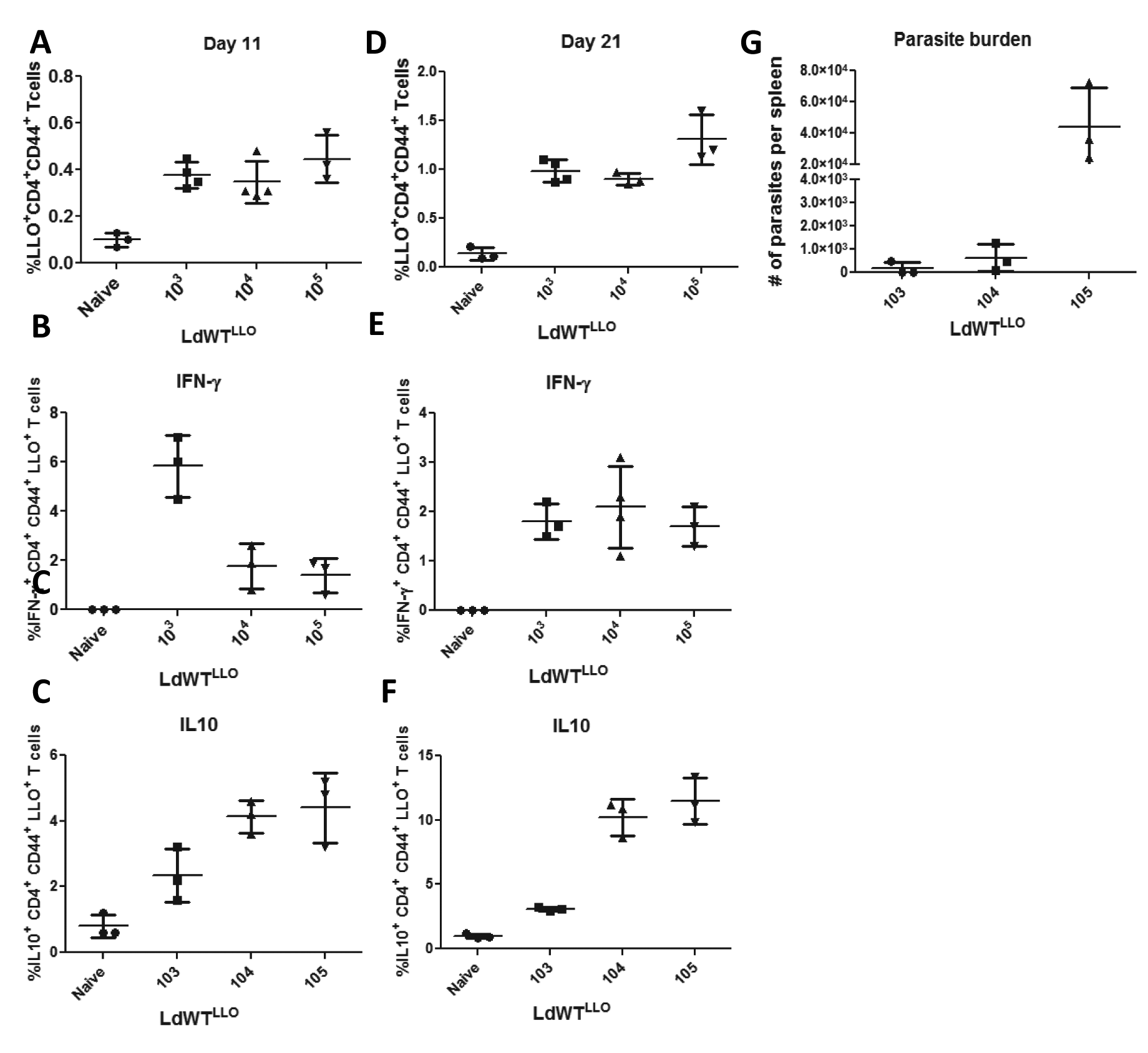

Supplement: Figure S3 — Enrichment and profiling of LLO+CD4+ T cells after a low dose LdWTLLO infection (additional data from repeat experiments). (A) Proliferation of LLO+CD4+ T cell populations at 11 days post infection. (B) IFN‐γ+CD4+LLO+ T cell population at 11 days post infection. (C) IL10+CD4+LLO+ T cell population at 11 days post infection. (D) Proliferation of LLO+CD4+ T cell populations at 21 days post infection. (E) IFN‐γ+CD4+LLO+ T cell population at 21 days post infection. (F) IL10+CD4+LLO+ T cell population at 21 days post infection. (G) Splenic parasite burden at day 21 post immunization (LdWTLLO). [file Image_3.TIF]
